# Magnetic field mapping of inaccessible regions using physics-informed neural networks

**DOI:** 10.1038/s41598-022-15777-4

**Published:** 2022-07-27

**Authors:** Umit H. Coskun, Bilgehan Sel, Brad Plaster

**Affiliations:** 1 Department of Physics and Astronomy, University of Kentucky, Lexington, KY 40506 USA; 2 The Bradley Department of Electrical and Computer Engineering, Virginia Tech, Blacksburg, VA 24061 USA

**Keywords:** Computational science, Techniques and instrumentation

## Abstract

A difficult problem concerns the determination of magnetic field components within an experimentally inaccessible region when direct field measurements are not feasible. In this paper, we propose a new method of accessing magnetic field components using non-disruptive magnetic field measurements on a surface enclosing the experimental region. Magnetic field components in the experimental region are predicted by solving a set of partial differential equations (Ampere’s law and Gauss’ law for magnetism) numerically with the aid of physics-informed neural networks (PINNs). Prediction errors due to noisy magnetic field measurements and small number of magnetic field measurements are regularized by the physics information term in the loss function. We benchmark our model by comparing it with an older method. The new method we present will be of broad interest to experiments requiring precise determination of magnetic field components, such as searches for the neutron electric dipole moment.

Magnetic field mapping is commonly used in many fields of science, medicine and technology such as particle accelerators, nuclear storage experiments^1–3^, cardiac beat detection^4^, magnetic resonance imaging (MRI)^5^ and magnetic indoor positioning systems (IPS)^6,7^. For example, in nuclear and particle physics experiments, with one example being the search for the neutron electric dipole moment, it is often crucial to measure and control the magnetic field components in the experimental region, because these experiments are typically sensitive to perturbations in magnetic fields. An undetected disturbance in a magnetic field may result in systematic uncertainties and cause a limitation for the precision of the measured quantities. To minimize systematic uncertainties, magnetic field components should be monitored in real-time and any unwanted field should be compensated during the operation time of the experiment. Real-time measurement of the magnetic field in an experimental region of space is not always practical or feasible. In most cases, the experimental region is not accessible due to a physical enclosure (e.g., a setup placed in a vacuum chamber), or it could be that placing a magnetic field sensor inside the experimental region is too disruptive to the system.

There exist several approaches in the literature that can be utilized to solve the problems stated above. For instance, Solin et al.^8^ make use of Gaussian processes (GPs) to interpolate/extrapolate ambient magnetic fields. They train the model using a data set collected by a magnetic field sensor at different locations of space and reconstruct the whole ambient magnetic field. Another method is proposed by Nouri et al.^9,10^. They introduced a non-disruptive magnetic field mapping method using exterior measurements at fixed locations and leverage the multipole expansion of the magnetic field vector. Expanding the magnetic field to some finite degree $n=N$, they provide a systematic way to optimize sensor locations and fit the unknown coefficients of the multipole expansion using the data from those exterior sensor measurements. This method is susceptible to noise in the data and, since the multipole expansion terms need to be picked to a specific field profile, the coefficients of the expansion terms are not regularized.

In this paper, we propose a robust way of predicting the magnetic field vector in the experimental region. In order to accomplish this, we utilize physics-informed neural networks (PINNs)^11^. PINNs propose a way to incorporate prior physical knowledge about the system in terms of its partial differential equations, into the deep neural networks while still being able to utilize their universal function approximator property. With PINNs, data and mathematical models of physics are combined in a smooth way, even in situations that are only partially understood, uncertain, and have a lot of dimensions. In noisy and high-dimensional situations, physics-informed learning blends data and mathematical models easily and can solve general inverse problems extremely successfully^12–14^. Unlike the method proposed in Refs.^9,10^, our method does not require prior knowledge of the multiple expansion terms to be fitted. This special type of neural network regularizes the output function (magnetic field prediction) during the training process by requiring the output to satisfy Maxwell’s equations, specifically Ampere’s law and Gauss’ law for magnetism.

## Methods

In this work, we are interested in predicting the magnetic field components inside a three dimensional space enclosed by an external surface *S* by utilizing the knowledge of the magnetic field in some number of locations on the surface *S*. Assuming there are no free currents, $\varvec{J}=0$, and no magnetization, $\varvec{M}=0$, in the region of interest, the partial differential equations that govern the static magnetic field are quite concise. They are

1 $$\begin{aligned} \varvec{\nabla \cdot B}= 0, \end{aligned}$$

and

2 $$\begin{aligned} \varvec{\nabla \times B}= 0. \end{aligned}$$

**Figure 1:**
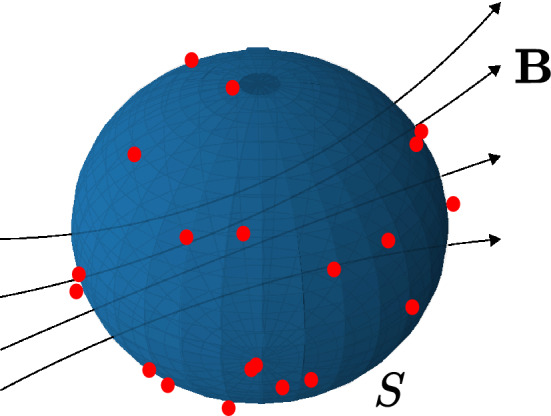
Magnetic field sensors (red dots) placed on a surface *S* to predict the magnetic field $\varvec{B}$ in the inner region.

Therefore, it is possible to find a magnetic field that satisfies (1) and (2) together with the knowledge of the magnetic field at some number of locations . We choose those locations on the surface of a closed region *S* of which we are interested in approximating the magnetic field inside (Fig. 1). Of course, according to the electromagnetism uniqueness theorem, having a finite number of data on a surface does not guarantee a unique solution to equations (1) and (2). However, as we demonstrate later in this paper, our results indicate that one can successfully approximate the true solution by having a sufficient number of data scattered on the surface.

### Review of the multipole expansion method

In this section, we review the field monitoring method described in Ref.^9^. Equation (2) indicates that the magnetic field vector can be written as a gradient of a scalar magnetic potential function

3 $$\begin{aligned} \varvec{B}= -\nabla \Phi _M(\varvec{r}). \end{aligned}$$

Substituting (3) into Eq. (1) tells us that the magnetic scalar potential satisfies Laplace’s equation

4 $$\begin{aligned} \nabla ^2\Phi _M=0, \end{aligned}$$

and the solution of Laplace’s equation in spherical coordinates is given by

5 $$\begin{aligned} \Phi _M(r,\theta ,\phi ) = \sum _l^\infty \sum _m^l r^l P^m_l(\cos \theta ) [a_{lm}\cos (m\phi )+b_{lm}\sin (m\phi )], \end{aligned}$$

where $P_l^m$ are the associated Legendre polynomials, and $a_{lm}$ and $b_{lm}$ are expansion coefficients. The magnetic field can be obtained by calculating the gradient of the magnetic scalar potential, $\varvec{B}= -\nabla \Phi _M(\varvec{r})$. Absorbing $a_{lm}$ and $b_{lm}$ in a coefficient $c_n$, we can write the magnetic field in a compact form

6 $$\begin{aligned} \varvec{B}(x,y,z) = \sum _n c_n \varvec{f}_n(x,y,z), \end{aligned}$$

where $\varvec{f}_n(x,y,z)$ are vector basis functions satisfying $\nabla \cdot \varvec{f}_n = 0$ and $\nabla \times \varvec{f}_n = 0$.

**Table 1:** Table of the *x*, *y* and *z* components of the basis vector function $\varvec{f}_n$ up to order $n=9$.

	$\varvec{f}_0$	$\varvec{f}_1$	$\varvec{f}_2$	$\varvec{f}_3$	$\varvec{f}_4$	$\varvec{f}_5$	$\varvec{f}_6$	$\varvec{f}_7$	$\varvec{f}_8$	$\varvec{f}_9$
*x*	0	$-1$	0	$-x$	$-3z$	0	6*x*	6*y*	$-3xz$	$\frac{3}{2}(3x^2+y^2-4z^2)$
*y*	0	0	$-1$	$-y$	0	$-3z$	$-6y$	6*x*	$-3yz$	3*xy*
*z*	1	0	0	2*z*	$-3x$	$-3y$	0	0	$-\frac{3}{2}(x^2+y^2-2z^2)$	$-12xz$

To illustrate, the first 10 $\varvec{f}_n$ basis vector functions are listed in Table 1. The right-hand side of the Eq. (6) is expanded to some finite order $n=N$ and the magnetic field vector inside the volume can be interpolated using linear regression techniques.

### Magnetic field prediction using PINNs

**Figure 2:**
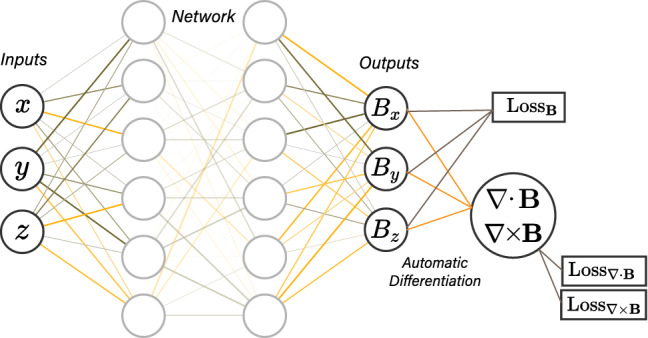
Network takes 3 inputs, (*x*, *y*, *z*) coordinates, and outputs the magnetic field $\varvec{B}$. Automatic differentiation is used to calculate the exact derivatives of the output $\varvec{B}$ with respect to the input parameters.

The exact values of the partial derivatives in (1) and (2) can be calculated by automatic differentiation^11^, which is implemented in some well-known machine learning libraries such as TensorFlow^15^ and PyTorch^16^. The neural network we train to approximate the magnetic field inside the region will have the structure as shown in Fig. 2. The hyperbolic tangent is used for the activation of each hidden layer. The other tested activation functions have not performed as well as the hyperbolic tangent for this network architecture. The number of hidden layers are chosen to be 4 and 8 with each having 32 or 64 neurons. The performance of these 4 different-sized networks are discussed later.

Then, the network can be trained by a combined loss function of data, curl and divergence losses

7 $$\begin{aligned} \text {Loss} = \text {Loss}_{\varvec{B}} + \lambda (\text {Loss}_{\varvec{\nabla \cdot B}} + \text {Loss}_{\varvec{\nabla \times B}}), \end{aligned}$$

where

8 $$\begin{aligned}&\text {Loss}_{\varvec{B}} := \frac{1}{N_{\varvec{B}}} \sum _{i=1}^{N_{\varvec{B}}} \Vert \varvec{B}(\varvec{r}_{\varvec{B}}^{i})-\varvec{B}_{s}(\varvec{r}_{\varvec{B}}^{i})\Vert ^2, \end{aligned}$$

9 $$\begin{aligned}&\text {Loss}_{\varvec{\nabla \cdot B}} := \frac{1}{N_f} \sum _{i=1}^{N_f} |\varvec{\nabla \cdot B}(\varvec{r}_{d}^{i})|^2, \end{aligned}$$

and

10 $$\begin{aligned} \text {Loss}_{\varvec{\nabla \times B}} := \frac{1}{N_f} \sum _{i=1}^{N_f} \Vert \varvec{\nabla \times B}(\varvec{r}_{d}^{i})\Vert ^2, \end{aligned}$$

where the points $\varvec{r}_{\varvec{B}}^{i}$ and $\varvec{r}_{d}^{i}$ denote the positions of the magnetic sensors and the collocation points, respectively. $N_{\varvec{B}}$ is the number of the magnetic field sensors, $N_f$ is the number of collocation points in the domain and $\varvec{B}_{s}$ is the measured magnetic field vector at $\varvec{r}_{d}^{i}$. The parameter $\lambda$ in Eq. (7) can be adjusted according to the performance of the network. The collocation points, $\varvec{r}_{d}^{i}$, in Eqs. (9) and (10) are sampled from the volume encapsulated by the surface *S* (Fig. 1) and can be chosen to be fixed throughout the training process^11^. However, randomly choosing collocation points in each epoch leads to a quicker convergence as well as more accurate results. This is partly due to being able to choose fewer number of collocation points, and since they are assigned randomly each iteration, they represent the domain better than any fixed collocation points scheme. The ADAM optimizer^17^, an adaptive method for gradient-based first-order optimization, is what we make use of in order to minimize the loss function 7. The general procedure for training is given in Algorithm 1.
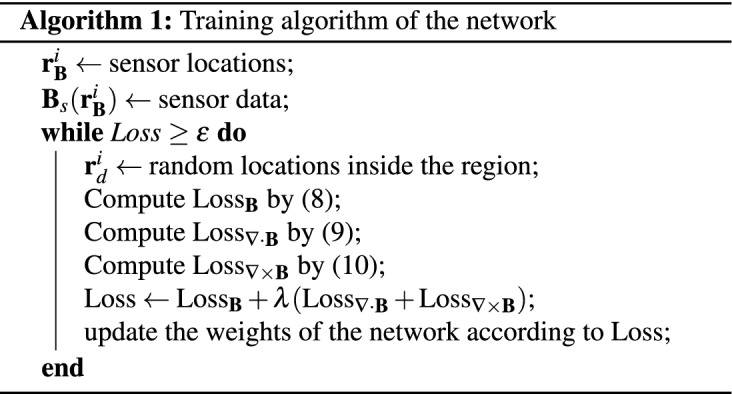


## Experiments

### Simulated experiment

In the following example, we will demonstrate the capability of our magnetic field prediction model by placing an arbitrary number of triple-axis magnetic sensors on the surface of a cube. Magnetic field sensors will be placed on the cube randomly and we will generate training and validation data by using Biot–Savart law for circular current loop(s). In the next section, we will give the analytical expression of the three dimensional magnetic field vector of a single circular current loop and then we will construct a higher order asymmetric magnetic field by placing multiple loops with different currents to benchmark our method on.

We begin by demonstrating the ability of our magnetic field reconstruction method by considering the magnetic field of a simple circular current loop (in arbitrary units). The magnetic field components of a circular current loop with radius *a* are given by^18,19^

11 $$\begin{aligned} \begin{aligned} B_x&= C\frac{xz}{2\alpha ^2 \rho ^2 \beta }\left[ (a^2 + r^2)E(k^2) - \alpha ^2 K(k^2)\right] \\ B_y&= \frac{y}{x}B_x\\ B_z&= C\frac{1}{2\alpha ^2 \beta }\left[ (a^2-r^2)E(k^2) + \alpha ^2 K(k^2)\right] \end{aligned} \end{aligned}$$

with

$$\begin{aligned} k^2 = \frac{4ar\sin \theta }{a^2+r^2+2ar\sin \theta }, \end{aligned}$$

where *E*(*k*) and *K*(*k*) are elliptic integrals, $\rho ^2 \equiv x^2 + y^2$ , $\alpha ^2 \equiv a^2 + r^2 -2a\rho$, $\beta ^2 \equiv a^2 + r^2 +2 a \rho$ and $r\equiv \sqrt{x^2+y^2+z^2}$ and $z = r\cos {\theta }$. In this work, we will work in arbitrary units by setting $C = 1$.

**Figure 3:**
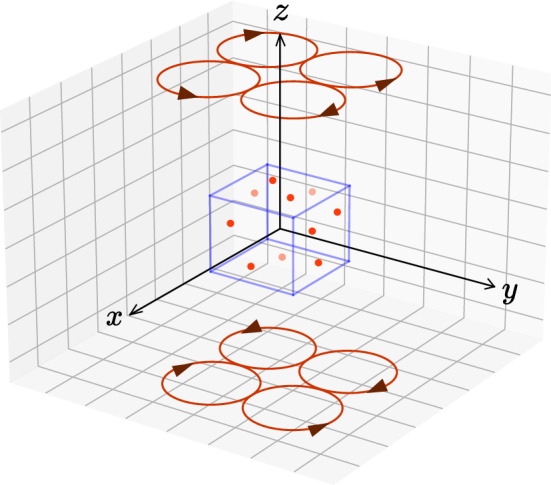
Configuration of the test model. Red circles: current loops, blue cube: cubical sensor array and red dot: triple-axis magnetic field sensors.

We want to show the potential of the network by comparing it to the multipole expansion method for various sensor counts and different types and levels of noises. To create a non-uniform higher order magnetic field, we positioned 8 circular loops of different current values at positions $(x=\pm 1.01, y=\pm 1,z=\pm 4)$ and the triple-axis magnetic sensors are placed randomly on the surface of a cube with side length $L=2$ centered at the origin. The configuration is illustrated in Fig. 3. Our goal is to predict the magnetic field in the inner region of the surface.

The number of hidden layers and neurons of the network characterizes the complexity of the function it can approximate. Having more hidden layers and neurons should not negatively affect the performance, training larger networks are slower and may require more care with initialization and regularization of the weights^20^. In this example, larger network sizes resulted in better performance as shown in Table 2 as expected. Models were trained for less than 2 min for all cases on an NVIDIA RTX 3080 GPU.

**Table 2:** Error between the predicted and exact magnetic field in vector norm for various sensor counts and network structures.

$N_{\varvec{B}}$	Network size			
	$4\times 32$	$4\times 64$	$8\times 32$	$8\times 64$
18	$1.9\times 10^{-2}$	$2.4\times 10^{-2}$	$2.2\times 10^{-2}$	$2.2\times 10^{-2}$
30	$8.5\times 10^{-3}$	$8.2\times 10^{-3}$	$7.9\times 10^{-3}$	$7.9\times 10^{-3}$
60	$1.9\times 10^{-3}$	$1.9\times 10^{-3}$	$1.9\times 10^{-3}$	$1.9\times 10^{-3}$
90	$1.7\times 10^{-3}$	$1.7\times 10^{-3}$	$1.7\times 10^{-3}$	$1.7\times 10^{-3}$

**Table 3:** Error measure in vector norm for the predicted and the other method’s different orders for various sensor counts.

Sensor count	PINN	4th order	6th order	8th order
18	$2.4\times 10^{-2}$	$2.0\times 10^{-1}$	8.1	25
30	$8.2\times 10^{-3}$	$1.5\times 10^{-1}$	$2.7\times 10^{-2}$	10
60	$2.9\times 10^{-3}$	$1.3\times 10^{-1}$	$1.6\times 10^{-2}$	$2.0\times 10^{-3}$
90	$1.8\times 10^{-3}$	$1.3\times 10^{-1}$	$1.4\times 10^{-2}$	$2.0\times 10^{-3}$

**Figure 4:**
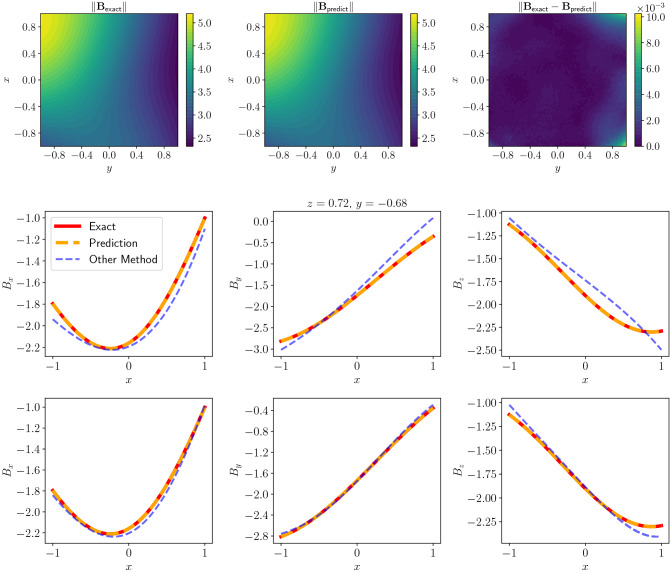
Top: Magnitude plot of the predicted magnetic field along with exact magnetic field and the error at snapshot $z=0.72$, middle: comparison of the predicted and the other method’s solutions’ *x*, *y*, *z* directions with 18 sensor data, bottom: comparison of the predicted and the other method’s solutions’ *x*, *y*, *z* directions with 30 sensor data.

**Figure 5:**
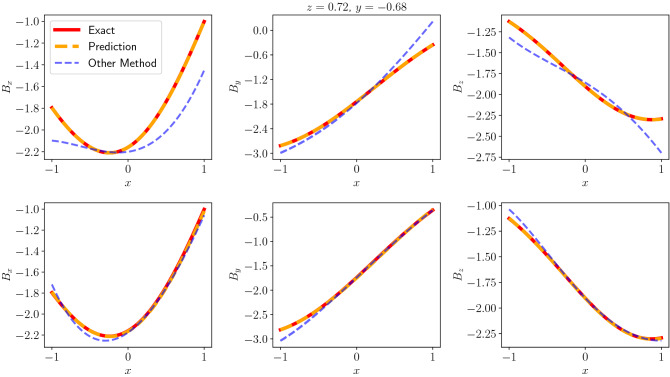
Top: Comparison of the predicted and the other method’s solutions’ *x*, *y*, *z* directions with 18 sensor data with Gaussian noise with $\sigma =1.0\times 10^{-2}$, Bottom: Comparison of the predicted and the other method’s solutions’ *x*, *y*, *z* directions with 30 sensor data with Gaussian noise with $\sigma =1.0\times 10^{-2}$.

Greater sensor counts gives more information about the magnetic field of the system, and we would expect the network to be able to use that information to predict magnetic field better. As shown in Table 2, having more sensory information has led to a better performance for all network structures. Moreover, fewer sensor counts has not led to a divergence from the exact magnetic field. This is not the case with the multipole expansion method as shown in Table 3. The other method seems to suffer with relatively few sensors and higher order versions overfit the sensor data. Decreasing the order in this case leads to better results but due to lower orders having fewer basis functions, the method is not able to predict the exact magnetic field as well as our network. This can also be seen in Figs. 4 and 5.

Performance of the network when Gaussian noise is introduced to the sensory information is given in Table 4. This noise has led to a further deterioration of the performance for the multipole expansion method. Our method has also been affected, however, performed better across various sensor counts.

**Table 4:** Error measure in vector norm for the predicted and the other method for various sensor counts with Gaussian noise added to the sensor data with standard deviations $\sigma =5.0\times 10^{-3}$ and $\sigma =1.0\times 10^{-2}$.

Sensor count	PINN (with noise)		Other method (with noise)	
	$5.0\times 10^{-3}$	$1.0\times 10^{-2}$	$5.0\times 10^{-3}$	$1.0\times 10^{-2}$
18	$2.6\times 10^{-2}$	$4.1\times 10^{-2}$	$2.0\times 10^{-1}$	$2.1\times 10^{-1}$
30	$1.2\times 10^{-2}$	$1.5\times 10^{-2}$	$2.9\times 10^{-2}$	$4.0\times 10^{-2}$
60	$5.0\times 10^{-3}$	$6.2\times 10^{-3}$	$8.0\times 10^{-3}$	$1.6\times 10^{-2}$
90	$4.5\times 10^{-3}$	$5.6\times 10^{-3}$	$8.0\times 10^{-3}$	$1.6\times 10^{-2}$

## Mapping the magnetic field of a square coil system

**Figure 6:**
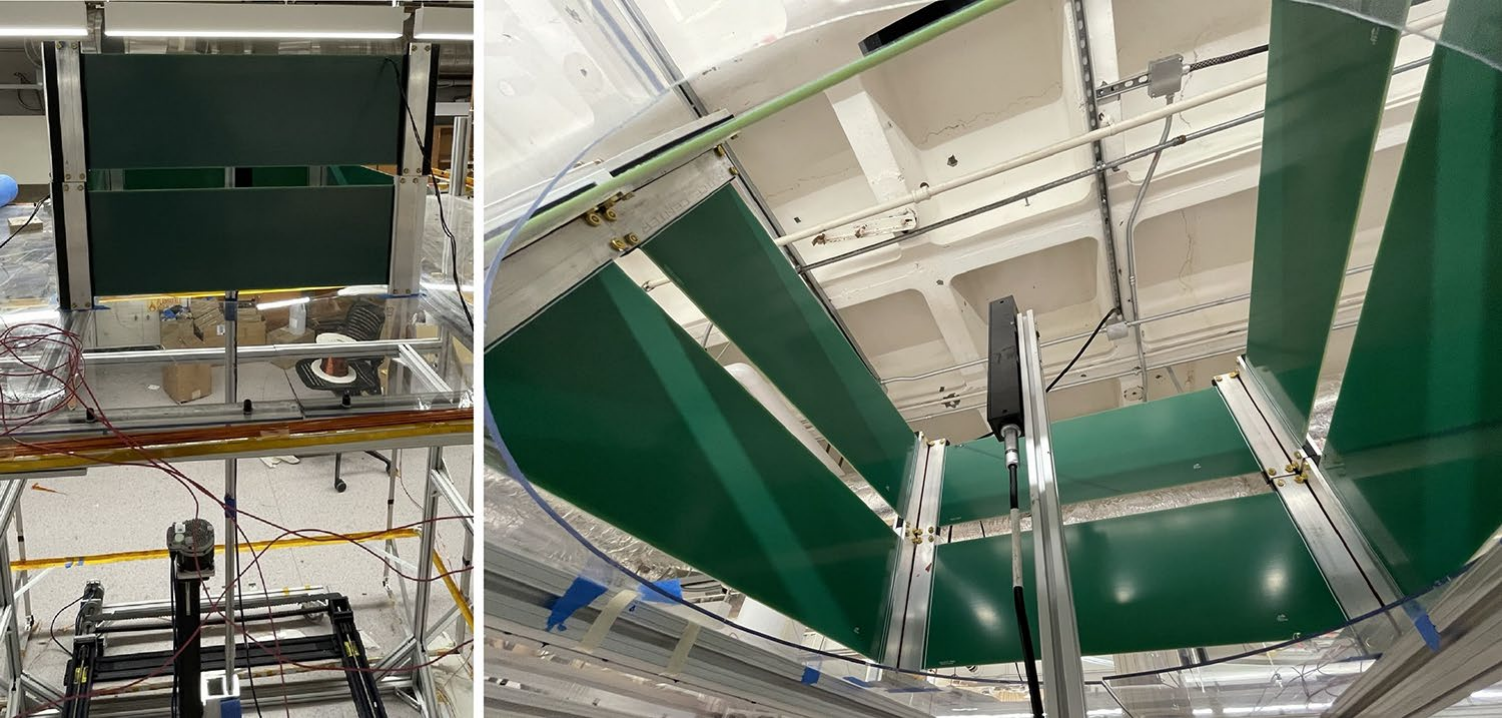
Setup of the experiment: Left: 3D magnetic field mapping system is sitting underneath the rectangular coils. Right: The triple-axis magnetometer and inside the coils.

To demonstrate our methodology using actual data, we conducted an experiment in which a Bartington triple axis magnetic field probe (Mag-13MS1000) was moved to the locations of the training data collection points. In order to generate a non-uniform magnetic field, two rectangular coils are stacked vertically and driven with different current magnitudes in the opposite directions (Fig. 6). Each face of the coils is a printed circuit board (PCB) with dimensions 55 cm $\times$ 16 cm containing 50 parallel line traces along the long side of the PCB. A current with magnitude 1 A flows counterclockwise and another current with magnitude 0.6 A flows clockwise in the top and bottom coils respectively.

**Figure 7:**
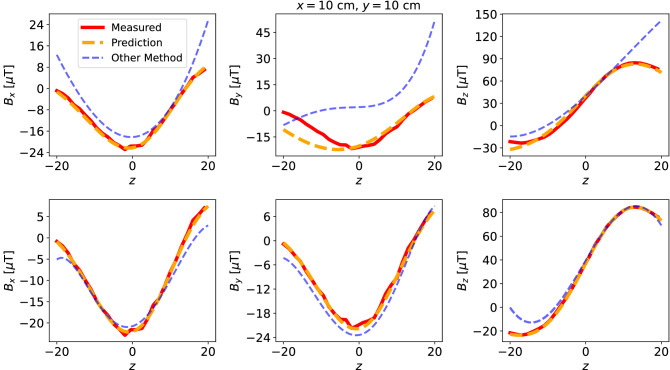
Comparison of the predicted and the other method’s solutions along $x =10$, $y=10$ axis. Top: 18 sensor data, bottom: 30 sensor data.

**Figure 8:**
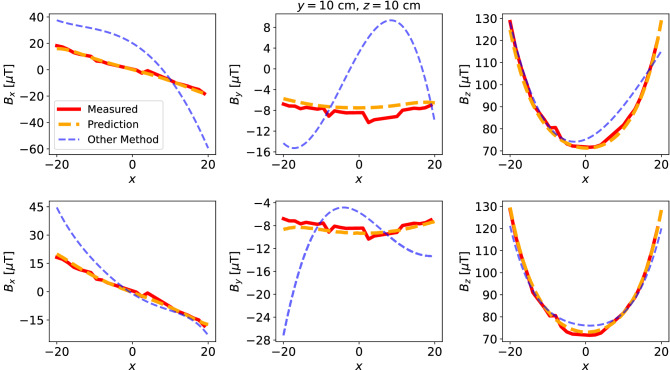
Comparison of the predicted and the other method’s solutions along $y =10$, $z=10$ axis. Top: 18 sensor data, bottom: 30 sensor data.

In order to isolate the field generated by the coils, at each measurement location, the data is collected as the difference between the sensor measurements with the coil turned on and off. Then, we trained the network for the magnetic field data collected using the magnetic mapping system. The training domain is chosen as a cube with 40 cm side length placed at the center of the coils. Performance benchmarks of our network and the other method on the measurement data is given in Figs. 7 and 8.

## Conclusions

In this study, we presented an efficient and practical method for mapping the magnetic field of inaccessible locations. We encoded previous knowledge from Maxwell’s equations for magnetostatics into a physics-informed neural network model for magnetic field prediction in regions where direct measurements are not possible.

We provided two experiments that proved the practicability of the proposed method. A simulated experiment proved the value of incorporating extra physics knowledge into the model. Mapping the magnetic field of a square coil system illustrated the effectiveness of the approximation technique in real world applications.

Our method compared with the multipole expansion method indicated better performance results across various sensor counts and noise levels both in simulation data and real world measurement data.

## Data Availability

The datasets generated and/or analysed during the current study are available in the Github repository, https://github.com/ucoskun/bmapping-pinn/tree/main/data.
